# Anoikis-related gene signatures in colorectal cancer: implications for cell differentiation, immune infiltration, and prognostic prediction

**DOI:** 10.1038/s41598-024-62370-y

**Published:** 2024-05-21

**Authors:** Taohui Ding, Zhao Shang, Hu Zhao, Renfeng Song, Jianyong Xiong, Chuan He, Dan Liu, Bo Yi

**Affiliations:** 1 School of Pharmacy, Jiangxi Medical College, Nanchang University, Nanchang, 330006 People’s Republic of China; 2 2nd Abdominal Surgery Department, Jiangxi Cancer Institute, Jiangxi Cancer Hospital, The Second Affiliated Hospital of Nanchang Medical College, Nanchang, 330029 Jiangxi People’s Republic of China; 3 Department of Digestive Oncology, Jiangxi Cancer Institute, Jiangxi Cancer Hospital, The Second Affiliated Hospital of Nanchang Medical College, Nanchang, 330029 Jiangxi People’s Republic of China

**Keywords:** Colorectal cancer, Anoikis, Gene signature, Prognostic model, Immunotherapy prediction, Data integration, Data mining, Machine learning, Predictive medicine

## Abstract

Colorectal cancer (CRC) is a malignant tumor originating from epithelial cells of the colon or rectum, and its invasion and metastasis could be regulated by anoikis. However, the key genes and pathways regulating anoikis in CRC are still unclear and require further research. The single cell transcriptome dataset GSE221575 of GEO database was downloaded and applied to cell subpopulation type identification, intercellular communication, pseudo time cell trajectory analysis, and receptor ligand expression analysis of CRC. Meanwhile, the RNA transcriptome dataset of TCGA, the GSE39582, GSE17536, and GSE17537 datasets of GEO were downloaded and merged into one bulk transcriptome dataset. The differentially expressed genes (DEGs) related to anoikis were extracted from these data sets, and key marker genes were obtained after feature selection. A clinical prognosis prediction model was constructed based on the marker genes and the predictive effect was analyzed. Subsequently, gene pathway analysis, immune infiltration analysis, immunosuppressive point analysis, drug sensitivity analysis, and immunotherapy efficacy based on the key marker genes were conducted for the model. In this study, we used single cell datasets to determine the anoikis activity of cells and analyzed the DEGs of cells based on the score to identify the genes involved in anoikis and extracted DEGs related to the disease from the transcriptome dataset. After dimensionality reduction selection, 7 marker genes were obtained, including *TIMP1, VEGFA, MYC, MSLN, EPHA2, ABHD2*, and *CD24*. The prognostic risk model scoring system built by these 7 genes, along with patient clinical data (age, tumor stage, grade), were incorporated to create a nomogram, which predicted the 1-, 3-, and 5-years survival of CRC with accuracy of 0.818, 0.821, and 0.824. By using the scoring system, the CRC samples were divided into high/low anoikis-related prognosis risk groups, there are significant differences in immune infiltration, distribution of immune checkpoints, sensitivity to chemotherapy drugs, and efficacy of immunotherapy between these two risk groups. Anoikis genes participate in the differentiation of colorectal cancer tumor cells, promote tumor development, and could predict the prognosis of colorectal cancer.

## Introduction

Colorectal cancer (CRC) is the malignant tumor derived from colon or rectal epithelial cells, which is common in the digestive field. According to reports, in the year 2017, the total number of CRC cases in the United States was 13, 5430, with approximately 50, 260 deaths and a mortality rate of 37.1%^1^. Although the overall incidence rate of CRC has declined in recent years, the number of people under 50 years old has increased by 2%^2^. Predictive analysis reveals that by the year 2030, the incidence rate of colon cancer aged 20–34 will increase by 90.0%, while that of rectal cancer will increase by 124.2%^3^. CRC has no characteristic symptoms in the early stage, while changes in stool characteristics and abdominal pain might occur in the middle or late stages^4^. Surgery is the preferred treatment for CRC patients with distant metastasis, but it has a cure rate of only 20–30%, and approximately 50–75% of patients experience recurrence after radical surgery^5^. In recent years, with the progress of surgical techniques, the emergence of new chemotherapy drugs, and the update of comprehensive treatment concepts, the survival rate and quality of life of CRC patients have been significantly improved^6^. However, for many patients, due to neglecting early symptoms, they might have already experienced distant metastasis or even lost the opportunity for surgery at their first visit, leading to poor prognosis^6^. Studies have shown that about 25% of patients experienced metastasis at their initial visit, while nearly half of patients ultimately experience liver metastasis, lung metastasis, bone metastasis or brain metastasis^7,8^. Therefore, the prevention of cancer metastasis is crucial for improving the prognosis of the disease.

The infiltration and metastasis of CRC are major factors influencing prognosis^9^. Currently, new molecules associated with rectal cancer metastasis have been discovered, including proliferation regulatory genes, apoptosis regulatory genes, adhesion molecules, growth factors, proteinases, and signaling transduction factors^10^. In recent years, it has been reported that resistance to anoikis apoptosis is associated with cancer metastasis^11^. Anoikis is a known form of programmed cell death caused by the loss or improper adhesion of cells to the extracellular matrix (ECM), playing a significant role in organism development and tissue homeostasis^12^. However, biochemical and molecular changes in the cellular environment lead to resistance to anoikis apoptosis in some tumor cells, allowing them to survive through anoikis signaling pathways. These changes are important factors in tumor cell invasion, metastasis, chemotherapy resistance, and recurrence^13^. Under normal circumstances, anoikis is activated when cells lose contact with the ECM, leading to programmed cell death to maintain homeostasis. However, some tumor cells survive due to their malignant potential to resist anoikis, allowing them to migrate to distant tissues through the circulatory system, leading to cancer cell invasion and dissemination^14^. Researchers have identified important proteins and pathways related to anoikis resistance in colorectal cancer. For example, a study by Fan et al.^12^ demonstrated that myosin-related kinase B can promote distant metastasis of cancer through anti-anoikis pathways, which is associated with poor prognosis. Another study indicated that the loss of anoikis in tumor cells is also associated with tumor progression and invasion^15^.

Single-cell RNA sequencing data analysis provides an unprecedented opportunity to explore the cellular subtypes and molecular characteristics of tumors, which could be an effective way to investigate the role of anoikis in CRC tumors^16^. Therefore, in this study, we used scRNA-seq data from CRC to identify anoikis-related genes (ARGs) and analyze their functions in promoting tumor invasion and metastasis, as well as their predictive ability for disease prognosis.

## Materials and methods

### Bulk transcriptome data acquisition and pre-processing

We obtained the RNA transcriptome data from The Cancer Genome Atlas (TCGA, https://portal.gdc.cancer.gov/) and the Gene Expression Omnibus (GEO, https://www.ncbi.nlm.nih.gov/geo/). The whole genome-wide expression profiles of CRC data in Transcripts Per Kilobase per Million (TPKM) format, along with the clinical annotations and simple nucleotide variation (SNV) data estimated by “VarScan2 Variant Aggregation and Masking” tool, were downloaded through the R package “TCGAbiolinks” (version 2.25.0)^17^ from TCGA. The TCGA-COAD dataset enrolls 521 samples, with tumor and healthy control samples (480/41), while the TCGA-READ dataset enrolls a total of 177 samples, with tumor and healthy control samples (167/10) respectively. We also downloaded the datasets of GSE39582 (tumor samples = 566, control samples = 19), GSE17536 (tumor samples = 177) and GSE17537 (tumor samples = 55) from GEO through the R package “GEOquery”. All the datasets mentioned above were merged to obtain a bulk transcriptome dataset. Batch effects sourced from technical biases were corrected by using the “ComBat” function in the package “sva”^18^. To examine the effect after correction, the principle component analysis (PCA) was introduced. We have also downloaded the CRC immunotherapy cohort GSE53127 from the GEO database and obtained the bladder cancer immunotherapy cohort IMvigor210 by using the IMvigor210CoreBiologies R package.

### Single-cell sequencing data download and processing

The single-cell raw dataset from GSE221575 of GEO containing 5 CRC samples were obtained, which was then normalized and batch-effect eliminated by using the functions in the R package “Seurat”^19^. Cell types were identified if their expressed genes (n) met 300 < n < 5000 with a proportion of mitochondrial genes < 10%. Genes were retained only if they expressed in more than one type of cell. Next, PCA was conducted and the shared nearest neighbor (SNN) algorithm was realized by calling the “FindClusters” function, to obtain the clusters based on the components with their resolution > 0.8. Different expressed genes (DEGs) of each cluster were identified by the “FindAllMarkers” function.

### The AUCell scores of the ARGs

The R package “AUCell” scores pathways for each cell and determines whether they are enriched by the gene set with a method of calculating their area under the curve (AUC)^20^. The initial anoikis genes (Supplement Table 1) were downloaded from Genecards database and ranked, which were then input to “AUCell_exploreThresholds” function for a threshold determination. Next, the t-distributed random neighbor embedding (t-SNE) was conducted for mapping the AUC scores and the results were visualized by the “ggplot2” package of R^21^.

### Pseudo-time analysis of cell trajectory

The analysis was processed by using the “Monocle 2” package^22^, which is a reverse graph embedding algorithm using DDTree as a tool^23^. It searches for highly dispersed and highly expressed specific genes by inferring the trajectory of anoikis activated cell subpopulations. To search for genes whose expression characteristics heavily depend on branching, we applied the branch expression analysis modeling (BEAM) method in the process of using “Monocle 2”^24^, and the results were displayed in a heat map.

### Cell communications and ligand-receptor expression analysis

Based on the CRC and control samples from the single-cell dataset, we initially applied the “CellChat”^25^ of the R package to identify common CellChat objects. Next, with “CellChatDB. Human” as the default preference data, we combined all CellChat objects by using the “mergeCellChat” function and called “netVisual_diffInteraction” to differentiate ligand receptor interactions between cells and visualize gene distribution.

### GO and KEGG pathway enrichment analysis

We performed differential expression analysis (DEA) between CRC and control samples from both the single-cell dataset and the bulk transcriptome dataset, and then intersected the DEGs with the known anoikis gene set to obtain the key anoikis-related differentially expressed genes (ARDEGs). The Gene Ontology (GO)^26^ annotates all the functions enriched by the specific gene set. The Kyoto Encyclopedia of Genes and Genomes (KEGG)^27^ is a bioinformatics resource for mining significantly pathways enriched in the gene list. The R package “clusterProfiler (version 4.2.2)”^28^ was applied to perform GO and KEGG enrichment analysis (*p* value < 0.05) on the key ARDEGs.

### Building and validation of a prognosis-prediction model

To investigate the prognosis-predicting value of the ARDEGs, we further conducted Univariate Cox analysis with the ARDEGs as the arguments, and the overall survival (OS) as the dependent variable, to calculate hazard ration (HR) of each gene. The genes with the *p*-value < 0.05 were identified as prognosis-related and selected for further analysis. The tumor samples with clinical information were randomly divided into a training cohort (n = 976) and a verifying cohort (n = 419) with a ratio 7:3. The least absolute shrinkage and selection operator (LASSO) analysis was performed on the prognosis-related genes to further select the characteristic genes and to develop a prognostic model. We chose the “glmnet” package^29^ to realize the process and derived a calculation formula of risk scores.

$${\text{risk}}{\text{score}} = \mathop \sum \limits_{i = 1}^{n} coef_{i} \times {\text{exp}}_{i}$$

The samples in the training cohort were scored and divided into two risk groups by using the median risk score as the dividing line, which are the low prognosis risk group (LRG) and the high prognosis risk group (HRG). Kaplan–Meier (KM) was to plot survival curves, and the difference of the two curves was compared and tested with log-rank test. Receiver operating characteristic (ROC) curves were introduced to determine the efficacy of the model with an AUC value of > 0.6 indicating a better performance. For a validation, the validation cohort was also divided into LRG and HRG for the same operation.

### Construction and verification of nomogram

The clinical information (age, gender and tumor stage) of patients was extracted as variables, along with the risk score to derive a regression model. We conducted the univariate/multivariate Cox regression analysis (UCRA/MCRA) on these variates and built a nomogram. The prediction efficacy was evaluated by the time-dependent ROC curves.

### GSEA

The Gene Set Enrichment Analysis (GSEA)^30^ determined if a gene set enriches in a specific pathway. We calculated the log2FC of the DEGs between LRG and HRG and ranked them, to be input into the “clusterProfiler” package for a 1,000 loop of calculations. The gene set “c2.cp.kegg.v7.5.1.symbols” from the Molecular Signatures Database (MSigDB)^31^ was collected as the reference.

### GSVA

The gene set variation analysis (GSVA) estimates the pathway activity between two gene expression matrixs. We set “c2.cp.kegg.v7.5.1.symbols” as the preference and used the package “GSVA” to perform the ananlysis^32^. A heat map was drawn to visualize the results.

### Immune infiltration analysis

The single-sample Gene Set Enrichment Analysis (ssGSEA)^33^ calculates the individual enrichment score for each pair of samples and gene sets, which represents the degree that a specific gene set is upregulated or downregulated in the sample. Based on the 28 types of immune cells downloaded from the Tumor and Immune System Interactions Database (TISIDB) (http://cis.hku.hk/TISIDB/index.php)^34^ (Supplement Table 2), the relative enrichment scores of immunocytes was quantified from each CRC sample’s gene expression profile. The immune cells infiltration level between the LRG and HRG were displayed with a box plot through the R package “ggplot2”.

### Expression of immune checkpoint genes

Several small molecules consist of the immune checkpoints (Supplement Table12). We compared well-known immune checkpoint genes (ICGs) between the two risks groups for their expression level.

#### Assessment of the drug susceptibility

Based on Genomics of Drug Sensitivity in Cancer (GDSC) (https://www.cancerrxgene.org/)^35^, we obtained the drugs with their half-maximal inhibitory concentration (IC50). The R package “oncoPredict”^36^ was introduced to predict the sensitivity of the potential therapeutic drugs in the two risk groups.

### Somatic mutation analysis

We usd the R package “maftools” to compare the mutation burden between the LRG and HRG^37^ and retained genes with mutations > 40. The mutation frequency between the two risk groups was also compared by using Wilcoxon tests.

### Tissue specimens

49 Fresh colon cancer tissue and adjacent normal tissue were collected from the Department of Abdominal Surgery at Jiangxi Cancer Hospital. None of the patients received any treatment before surgery, and all patients signed informed consent forms provided by Jiangxi Cancer Hospital. The primary tumor area and the surgical margin tissue with normal morphology were immediately isolated from each patient by an experienced pathologist and stored in liquid nitrogen until use. This study was approved by the Jiangxi Cancer Hospital Hospital (2022KY293).

### RNA extraction and RT-qPCR

Target gene mRNA expression in tissues of colorectal cancer patients were examined relative to GAPDH.Total RNA was extracted from Normal and cancerous tissues of the human colon by the RNAiso Plus (T9180, takara, Japan).And the concentration of total RNA was measured using Ultramicro protein nucleic acid analyzer (BioDropμlite + , BioDrop, England). RNA integrity was detected by agarose gel electrophoresis. RNA was reverse-transcribed to cDNA using a PrimeScript RT reverse transcription kit (Takara, Japan), Then qRT-PCR was carried out by using TB Green® Premix Ex Taq™II to evaluate the expression abundance of mRNA. Primers were designed and synthesized by Shanghai Shenggong Biology. GAPDH was used as the normalization control, and the 2-ΔΔCT. The primer sequences were as follows: VEGFA Forward Sequence 5′-3′: CTGGAGCGTGTACGTTGGT and Reverse Sequence 5′-3′: TTTAACTCAAGCTGCCTCGC; TIMP1Forward Sequence 5′-3′: CTTCTGCAATTCCGACCTCGT and Reverse Sequence 5′-3′:ACGCTGGTATAAGGTGGTCTG; MYC Forward Sequence 5′-3′:TGGAAAACCAGCCTCCCG and Reverse Sequence 5′-3′: TTCTCCTCCTCGTCGCAGTA; ABHD2 Forward Sequence 5′-3′: AATCACACGCAGGCACAGAT and Reverse Sequence 5′-3′: AATCACACGCAGGCACAGAT; EPHA2 Forward Sequence 5′-3′: TGGCTCACACACCCGTATG and Reverse Sequence 5′-3′: GTCGCCAGACATCACGTTG;MSLN Forward Sequence 5′-3′: TGGCCTTGGCACAGAAGAAT and Reverse Sequence 5′-3′: GAACGCATCTGGGTTGAGGA; GAPDH Forward Sequence 5′-3′: CCTGGTATGACAACGAATTTG and Reverse Sequence 5′-3′: CAGTGAGGGTCTCTCTCTTCC. GAPDH served as an internal control.

### Statistical methods

The Wilcoxon rank-sum test was used to test the difference of two groups of continuous variables. A two-sided *p*-value < 0.05 was treated significant for all comparing cases. All analyses were done in R software (version 4.1.2).

### Ethics approval and consent to participate

The study was approved by the Ethics Review Committee of Nanchang Medical College, and informed 49 consent was obtained from all patients before the study.

## Results

### The flow chart

**Figure 1:**
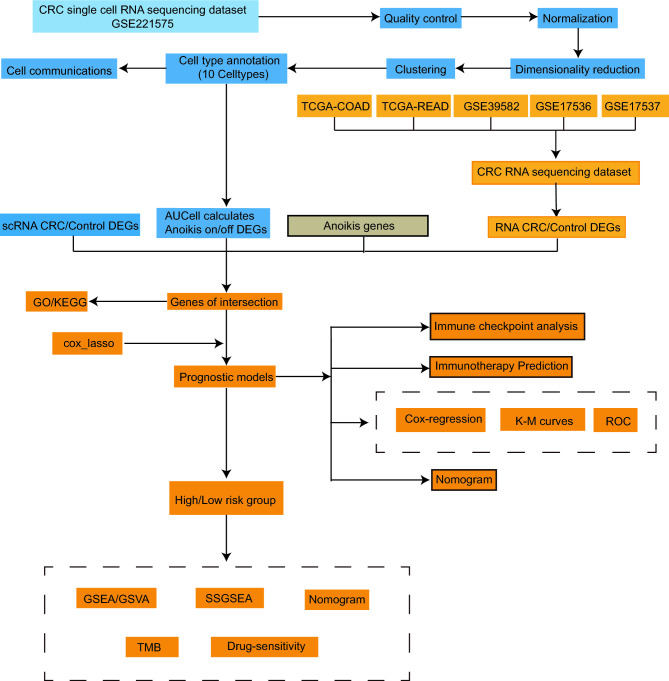
The workflow of the study.

The workflow chart of this study was depicted in Fig. 1.

### Single-cell sequencing analysis

**Figure 2:**
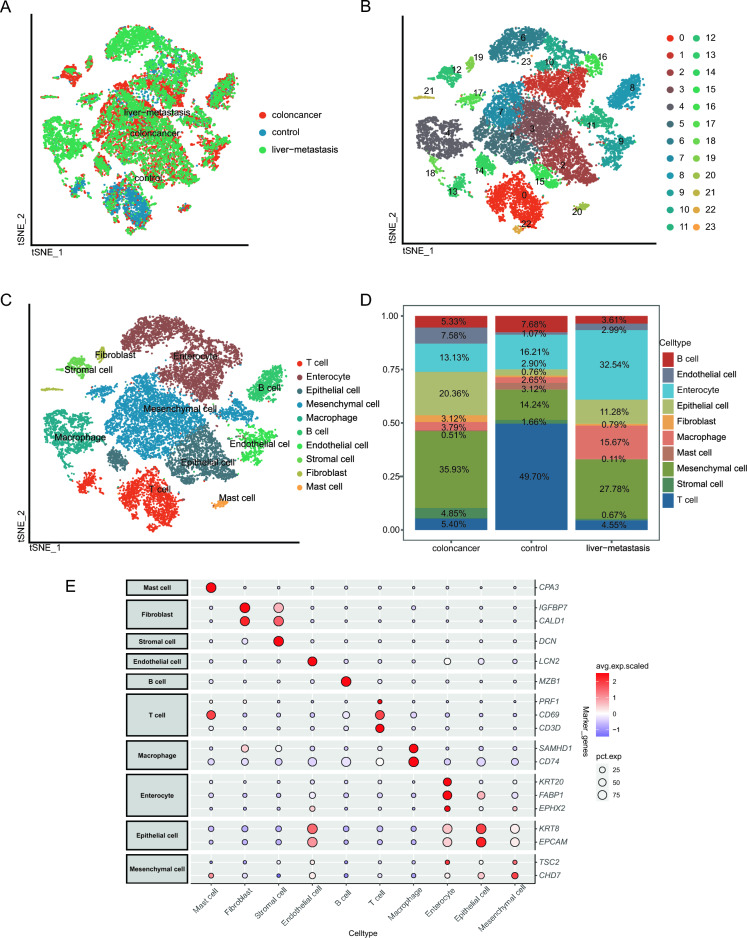
Identification of cell subgroups and marker genes in GESE221575. (**A**) tSNE map of CRC, control group and liver metastasis samples from colorectal cancer. (**B**) tSNE map of cell subtypes. (**C**) tSNE map of the annotation results of CRC cell subgroups. (**D**) Cumulative histogram of cell types in patients with CRC, control group and liver metastasis samples. (**E**) Expression profiles of the marker genes in each cell type. tSNE, t-distributed random neighbor embedding; CRC, colorectal cancer.

We initially analyzed the cell subtypes of CRC in the GSE221575 dataset and obtained a total of 19,711 cells from cells identification (Fig. 2A). Next, we obtained 24 clusters after executing the clustering algorithm (Fig. 2B). Based on the genetic characteristics of each cluster, a total of 10 cell types were found and showed (Fig. 2C). The proportions of cell types in each sample are shown in Fig. 2D. The specific genes for each cell type were visualized by dot plot (Fig. 2E). We found that the proportion of T cells in the colon cancer group or the liver-metastasis group is significantly lower than that in the control group, whereas the proportions of Mesenchymal cell in the colon cancer and Enterocyte in the liver-metastasis were higher than that in the other two groups respectively.

### Identification of gene activity of anoikis

**Figure 3:**
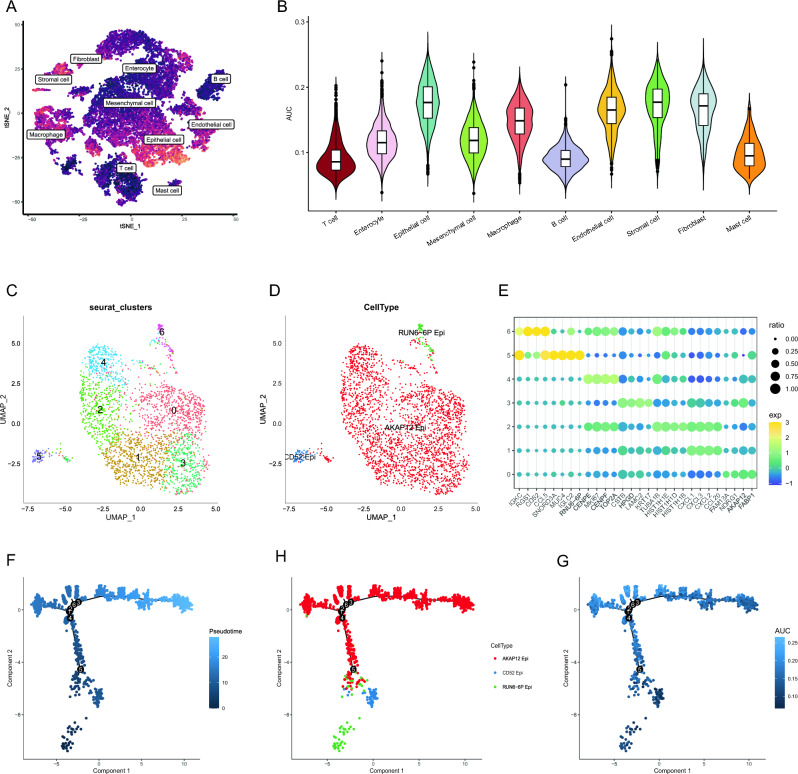
Transcriptional trajectory analysis revealed an association between anoikis and epithelial differentiation. (**A**) tSNE graph. The anoikis activity resembles in brighter color. (**B**) Violin plot of anoikis AUC value in different cell types. (**C**) UMAP plot showing the clustering distribution of epithelial cells. (**D**) Annotation results of epithelial cells. (**E**) Bubble plots of marker genes for different subgroups of epithelial cells. (**F**) Pseudotime plot of epithelial cells. (**H**) Pseudotime plots of different epithelial cells cell types. (**G**) Pseudotime plot of the anoikis activity.

We defined the cell population with AUC value greater than 0.2 as the cell population with high anoikis activity, and the cell population with AUC value less than 0.2 as the cell population with low anoikis activity. In addition, as shown in the Fig. 3A,B, epithelial cells, fibroblasts, and stromal cells have higher anoikis activity.

### Pseudo-time trajectories analysis

To elucidate the role of anoikis genes in the progression of colorectal cancer (CRC), we constructed a transcriptional trajectory to identify the underlying gene expression programs driving CRC progression. The transcriptional states in the trajectory revealed the process of differentiation. Epithelial cells are the main malignant cell source in CRC. We initially analyzed the epithelial cells, and after extracting and re-clustering them, we performed differential gene expression analysis on seven subgroups of cells. We found that cells in groups 0, 1, 2, 3, and 4 exhibited similar gene expression patterns, while they differed from cells in groups 5 and 6 (Fig. 3C,E). Therefore, we divided the epithelial cells into three cell groups: AKAP12 epithelial cells, CD52 epithelial cells, and RUN6-6P epithelial cells (Fig. 3D). Subsequently, we conducted pseudotime analysis on the epithelial cells. From the graph, it can be observed that CD52 epithelial cells and RUN6-6P epithelial cells are located at the initial part of the trajectory, while AKAP12 epithelial cells are located at the terminal part (Fig. 3F–H). Furthermore, we discovered that the anoikis activity gradually increased as the epithelial cells differentiated (Fig. 3G). Therefore, we believe that anoikis may be involved in the differentiation of tumor cells.

Furthermore, to explore the role of anoikis genes in the differentiation of mesenchymal cells, fibroblasts, and stromal cells, we performed pseudotime analysis on them. The results showed that as pseudotime advanced, the anoikis activity of stromal cells also gradually increased (Supplement Fig. 1A,B). Additionally, mesenchymal cells were located at the initial part of the trajectory, while fibroblasts and stromal cells were located at the terminal part (Supplement Fig. 1C–F), indicating that the differentiation of mesenchymal cells into fibroblasts and stromal cells is accompanied by an increase in anoikis activity. In order to elucidate the molecular basis of stromal cell differentiation, we explored the gene functions that determine the fate of CRC stromal cells. Genes highly expressed before branching mainly enriched in biological processes such as CXCR chemokine receptor binding and chemokine activity. Genes enriched in Golgi lumen, bicellular tight junction and tight junction pathways were highly expressed in cell fate 2, while genes enriched in collagen-containing extracellular matrix, cytosolic ribosome, focal adhesion, and cell-substrate junction-related pathways were highly expressed in cell fate 1 (Supplement Fig. 1G). In conclusion, we believe that anoikis genes significantly contribute to the processes of epithelial cell differentiation in CRC, as well as the differentiation of mesenchymal cells into fibroblasts and stromal cells.

### Cellular communication patterns in CRC microenvironment

**Figure 4:**
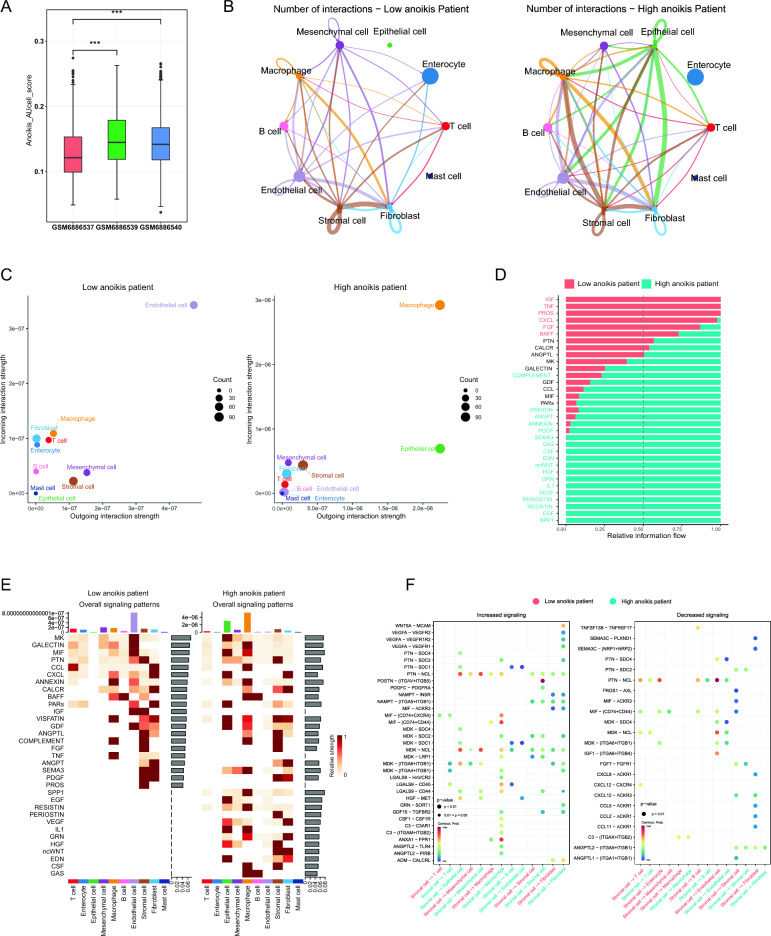
Overall pattern of intercellular communication analysis. (**A**) The box plots display the differences in anoikis activity among three sample groups, including CRC sample from non-metastatic patients (GSM688537), metastatic tumor patients (GSM688539) and metastatic tumor samples (GSM688540). (**B**) Network plots show the interaction numbers among cell types in the Low anoikis patient and High anoikis patient. (**C**) Bubble plots illustrate the internal and external cell interaction strength in the Low anoikis patient and High anoikis patient. (**D**) Bar plots demonstrate the relative information strength in the Low anoikis patient and High anoikis patient. (**E**) Heatmap of the overall signaling pathways in Low anoikis patient and High anoikis patient. **(F)** Significantly increased and reduced ligand receptor pairs in the Low anoikis patient and High anoikis patient.

Due to the association between anoikis and tumor invasion and metastasis, we analyzed the overall differences in anoikis activity between tumor samples from non-metastatic patients and samples from primary tumors and metastases in patients. We found that the anoikis activity in the two samples from metastatic CRC (colorectal cancer) patients was higher than that in non-metastatic CRC patients (Fig. 4A). Therefore, we divided the patients into a high anoikis activity group and a low anoikis activity group and used the "Cellchat" R package to uncover the differences in communication and interactions between the two groups. The high anoikis activity group showed a higher overall number of interactions compared to CRC non-metastatic patients (Fig. 4B). Additionally, in the high anoikis activity group, we observed a significant increase in both outward and inward interactions between epithelial cells and macrophages (Fig. 4C). Furthermore, we compared the signaling patterns between the high anoikis activity group and the low anoikis activity group. We found that the high anoikis activity group exhibited stronger signals in signaling patterns represented by genes such as SPP1, EGF, RESISTIN, PERIOSTIN, and VEG compared to the low anoikis activity group (Fig. 4D). Moreover, we also discovered a significant enhancement in signaling patterns represented by genes MK, MIF, CXCL, EGF, and IL1 in epithelial cells within the high anoikis activity group (Fig. 4E). Next, we found that, compared to the low anoikis activity group, the most significant changes in the number of ligand-receptor pairs occurred between stromal cells and epithelial cells, as well as between stromal cells and macrophages in the high anoikis activity group (Fig. 4F). Additionally, we found that in the high anoikis activity group, the signaling pathways represented by the PTN and MDK genes between stromal cells and epithelial cells were significantly enhanced, as well as the signaling pathways represented by the C3, ANXA1, and MIF genes between stromal cells and macrophages (Fig. 4F). These results suggest that anoikis genes may significantly influence the Cellular communication within the tumor.

### Enrichment analysis of ARDEGs in CRC

**Figure 5:**
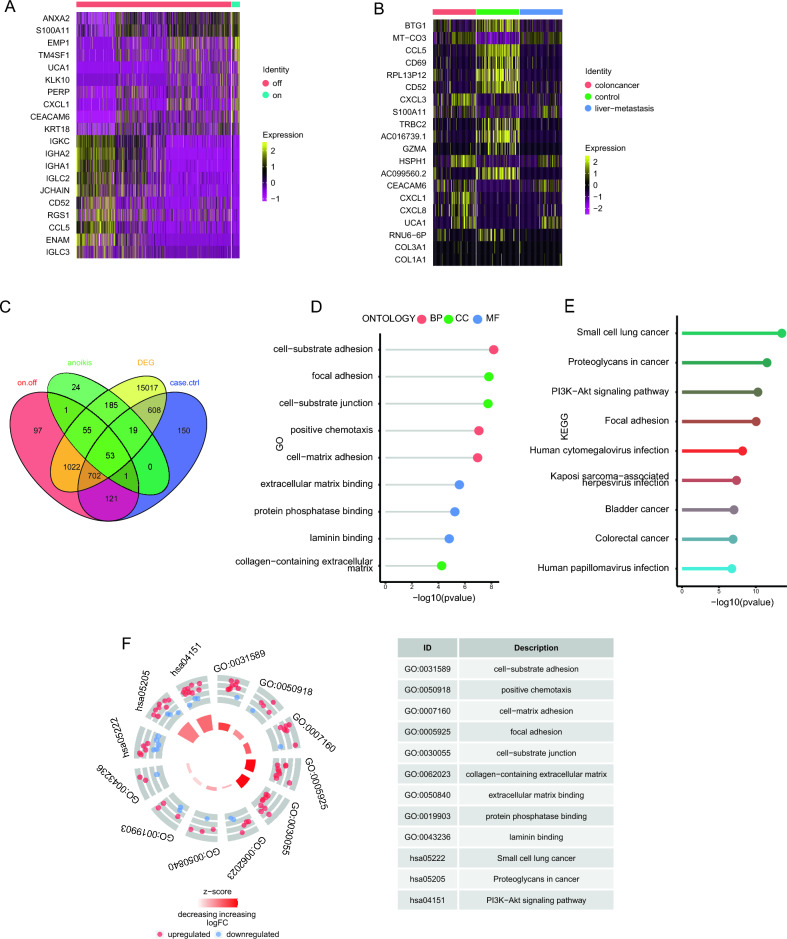
Enrichment analysis of DEGs related to anoikis activity in CRC. (**A**) The heatmap shows the significantly DEGs in anoikis active cells of CRC. (**B**) The heatmap shows the significantly DEGs between CRC and controls in single-cell dataset. (**C**) The Venn diagram of the key genes. (**D**) Lollipop chart shows GO enrichment analysis results. (**E**) Lollipop chart shows KEGG enrichment analysis results. (**F**) Circle diagram shows GO and KEGG enrichment results of intersection genes. GO, Gene Ontology; KEGG, Kyoto Encyclopedia of Genes and Genomes.

A total of 2052 DEGs were identified between the anoikis active and inactive cells, with 1767 genes up-regulated and 285 genes down-regulated (Supplement Table 3). The top 10 up-regulated genes (*ANXA 2, S100A11, EMP 1, TM4SF1, UCA 1, KLK 10, PERP, CXCL 1, A, CEACAM6, KRT 18*) and the top 10 down-regulated genes (*IGKC, IGHA 2, IGHA 1, IGLC 2, JCHAIN, CD52, RGS 1, CCL 5, ENAM, IGLC 3*) were shown by heat map (Fig. 5A). We also performed DEA between CRC samples and healthy controls for the single-cell dataset and the bulk transcriptome datasets separately. A total of 1654 DEGs were identified in the single-cell dataset with 747 genes up-regulated and 907 genes downregulated (Supplement Table 4). The top 10 up-regulated genes (*MT-CO3, CXCL 3, S100A11, HSPH 1, CEACAM6, CXCL 1, CXCL 1, CXCL 8, UCA 1, COL3A1, COL1A1*) and the top 10 down-regulated genes (*BTG 1, CCL5, CD69, RPL13P12, CD52, TRBC2, AC016739.1, GZMA, AC099560.2, RNU 6-6P*) were shown by heatmap (Fig. 5B). A total of 17,661 DEGs were identified in the bulk transcriptome dataset (Supplement Table 5). All the entry criteria of the DEGs were adjusted *p*-value < 0.05 and | Log2 fold change |> 0.25. A total of 53 key ARDEGs were obtained after intersecting the three sets of DEGs and a Venn plot was shown (Fig. 5C).

The GO analysis showed that these genes are enriched in cell − substrate adhesion, positive chemotaxis, cell − matrix adhesion (BP), focal adhesion, cell − substrate junction, collagen − containing extracellular matrix (CC), extracellular matrix binding, protein phosphatase binding, laminin binding (MF) (Fig. 5D, Supplement Table 6).

The KEGG analysis showed that these genes are enriched in pathways as small cell lung cancer, Proteoglycans in cancer, PI3K − Akt signaling pathway, Focal adhesion, Human cytomegalovirus infection, Kaposi sarcoma − associated herpesvirus infection, Bladder cancer, Colorectal cancer, Human papillomavirus infection (Fig. 5E, Supplement Table 7).

In addition, we calculated the z-scores of each item of GO and KEGG with their logFC ranked and plot a circle graph (Fig. 5F). The key ARDEGs were mainly enriched in cell − substrate junction, focal adhesion, Proteoglycans in cancer and PI3K − Akt signaling pathway.

### Construction and validation of a prognostic risk model

**Figure 6:**
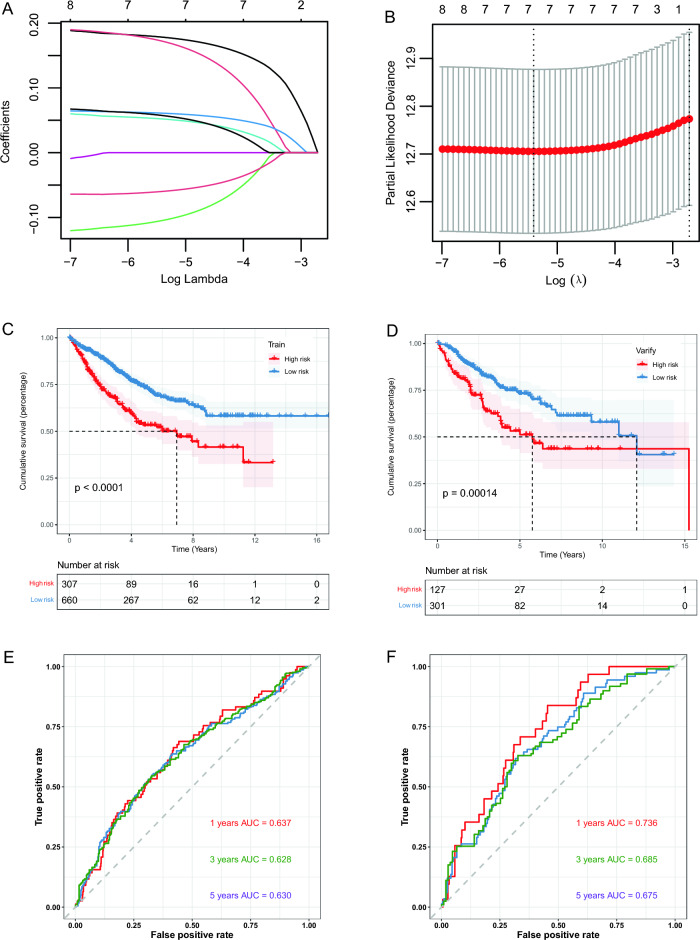
LASSO analysis. (**A**) Change trajectory of LASSO regression. (**B**) Confidential interval of each lambda. (**C**) The survival curve of the two risk groups in the training cohort and (**D**) in the validation cohort. Red for HRG, blue for LRG. (**E**) 1-, 3-, and 5-years time-dependent ROC curves of models for training cohort. (**F**) For the validation cohort. LASSO, least absolute shrinkage and selection operator.

We performed UCRA to identify the signature genes in the 53 key genes. A total of 9 genes were identified with *p* < 0.05, indicating association with prognosis (Supplement Table 8). LASSO regression analysis on the training set with the initial seed = 92 chose 7 signature genes out of the 9 genes (Fig. 6A,B, Supplement Table 9), along with a prognostic risk model built by these genes and their covariates. The 7 signature genes were *TIMP1, VEGFA, MYC, MSLN, EPHA2, ABHD2,* and *CD24.* A risk scoring system was also derived for the division of LRG/HRG. KM survival curves comparing the two groups in training cohort (Fig. 6C), and also in the validation cohort (Fig. 6D). The results showed a significant worse prognosis in the HRG compared to that in the LRG for both the cohorts. The ROC curves showed AUC values of 1-,3-,5-years survival as 0.637, 0.628, and 0.630 in the training cohort (Fig. 6E), while 0.736, 0.685, and 0.675 in the validation cohort (Fig. 6F).

### GSEA and GSVA

**Figure 7:**
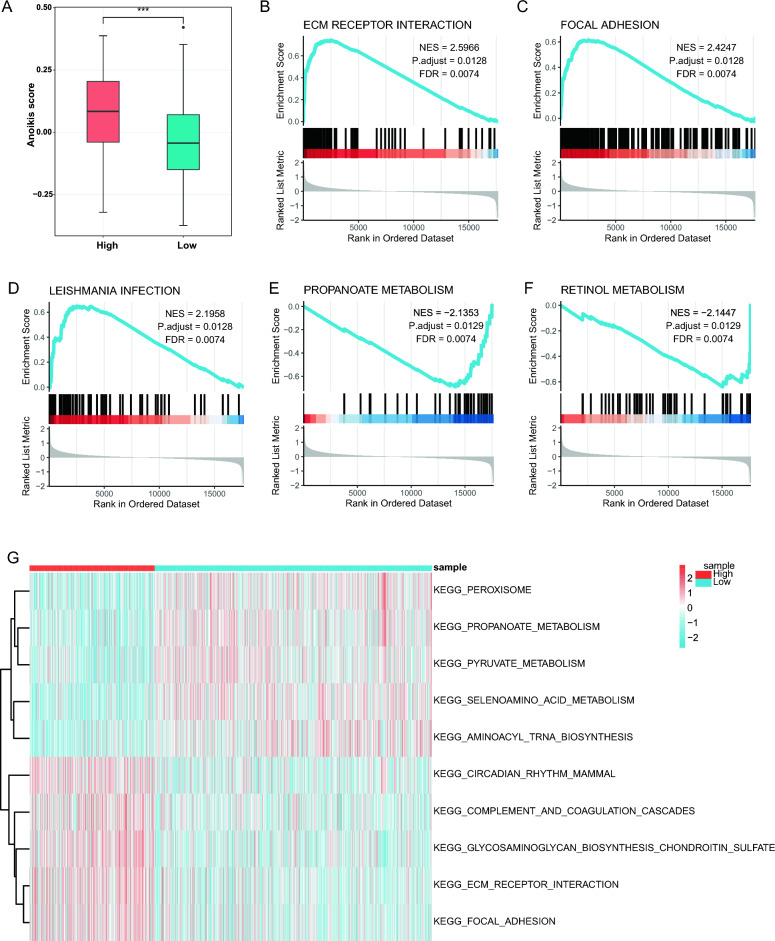
GSEA and GSVA results. (**A**) Anoikis scores between HRG and LRG. Results of enrichment analysis from GSEA: ecm receptor interaction (**B**), focal adhesion (**C**), leishmania infection (**D**), propanoate metabolism (**E**), retinol metabolism (**F**). (**G**)GSVA of significantly enriched pathways. GSEA, Gene set enrichment analysis; GSVA, gene set variation analysis.

Based on the high expression of seven marker genes in cells with high anoikis activity in the single-cell dataset (Supplement Table 3), as well as the high expression of these genes in tumor tissues in the bulk transcriptome data(Supplement Table 5), the GSVA method was employed to determine the anoikis score for each patient. The analysis revealed that the anoikis score was markedly elevated in the high-risk group compared to the low-risk group, as illustrated in Fig. 7A. Building upon this observation, we conducted a differential gene expression analysis based on risk stratification within the bulk transcriptome data to investigate the underlying mechanisms behind these gene expression differences. Subsequently, we performed GSEA analysis on the bulk transcriptome dataset and selected the most significantly enriched signaling pathways based on their normalized enrichment score (NES) (Supplement Table 10). We identified ecm receptor interaction (NES = 2.5966, adjusted P = 0.0128, FDR = 0.0074, Fig. 7B), focal adhesion (NES = 2.4247, adjusted P = 0.0128, FDR = 0.0074, Fig. 7C), leishmania infection (NES = 2.1958, adjusted P = 0.0128, FDR = 0.0074, Fig. 7D), propanoate metabolism (NES = -2.1353, adjusted P = 0.0129, FDR = 0.0074, Fig. 7E), retinol metabolism (NES = -2.1447, adjusted P = 0.0129, FDR = 0.0074, Fig. 7F) were significantly enriched in Colorectal cancer.

In addition, we also conducted GSVA analysis, and obtained 5 pathways with the most significant difference between the two risk groups and a heatmap was plotted (Supplement Table 11, Fig. 7G).

### Immune infiltration analysis

**Figure 8:**
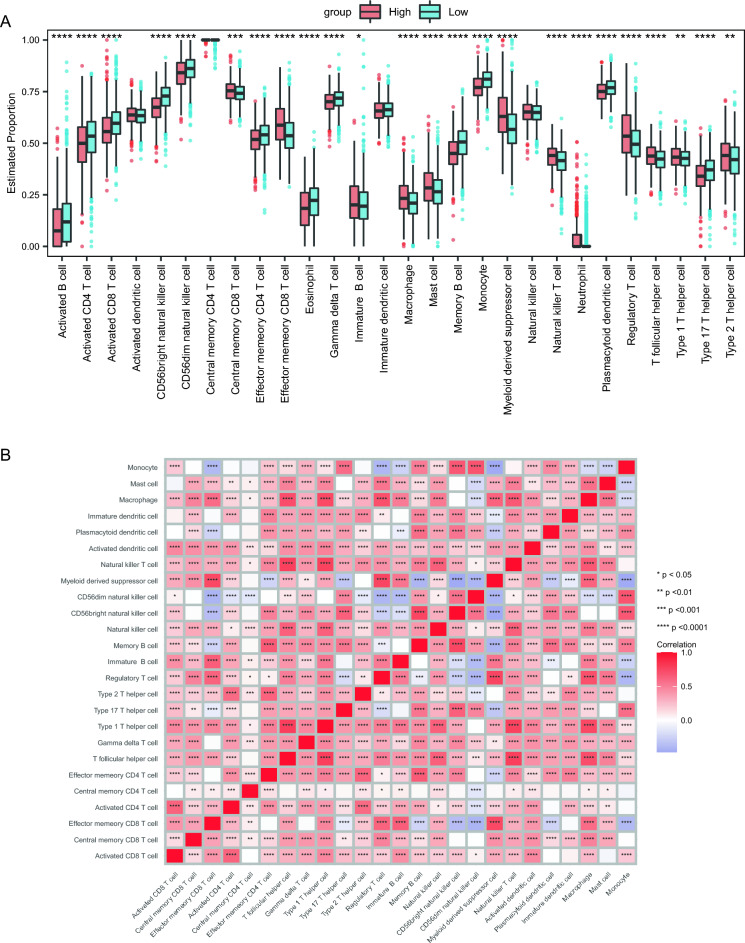
Immune infiltration between HRG and LRG. (**A**) Boxplot of the estimated proportion. (**B**) Correlation among immune cells. Asterisks represented p-value (****p < 0.0001, ***p < 0.001, **p < 0.01, *p < 0.05).

We further surveyed the infiltration levels of 28 immune cell types among the two risk groups using the ssGSEA method and found Activated B cell, Activated CD4 T cell, and Activated CD8 T cell showed significant differences among the two risk groups (*p* value < 0.05, Fig. 8A). The correlation analysis of the immune cells showed that the majority of them were positively correlated with each other, whereas some were negatively correlated with each other, such as Myeloid derived suppressor cell and Effector memeory CD4 T cell (Fig. 8B).

The correlations between 7 key genes and the immune cells were also calculated (Supplement Fig. 2A–I). We found gene *MYC* is significantly associated with CD56bright natural killer cell (R = 0.3905, And *p* < 0.001) and Memory B cell (R = 0.4238, *p* < 0.001) (Supplement Fig. 2A,B); gene *TIMP1* is significantly associated with Effector memeory CD8 T cell (R = 0.3823, *p* < 0.001), Macrophage (R = 0.4273, *p* < 0.001), Mast cell (R = 0.3233, *p* < 0.001), Monocyte (R = − 0.3921, *p* < 0.001), Myeloid derived suppressor cell (R = 0.4864, *p* < 0.001), Natural killer cell (R = 0.3399, *p* < 0.001) and Regulatory T cell (R = 0.4865, *p* < 0.001) (Supplement Fig. 2C–I).

### Construction and verification of nomogram

**Figure 9:**
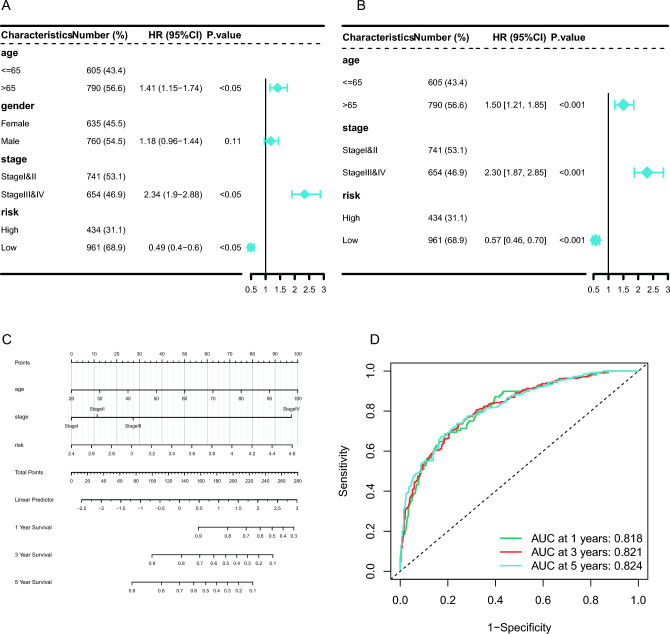
Construction and verification of nomogram. (**A**) Forest map of univariate analysis for the clinicopathologic characteristics and risk in CRC cohort. (**B**) Forest map of multivariate analysis for the clinicopathologic characteristics and risk in CRC cohort. (**C**) A nomogram was established to predict the prognostic of CRC patients. (**D**) 1-, 3-, and 5-years time-dependent ROC curves.

The UCRA/MCRA were performed on the risk score variate, along with the clinical characteristics of age, stage, and grade, to determine if these variates could be the prognosis factors. The results showed that risk score could act as an independent factor for prognosis (Fig. 9A,B). The results of the UCRA were used to build the nomogram (Fig. 9C). The ROC curve showed good AUC values of 1-, 3-, and 5-years survival, 0.818, 0.821, and 0.824, respectively (Fig. 9D).

### TMB and drug susceptibility analysis

**Figure 10:**
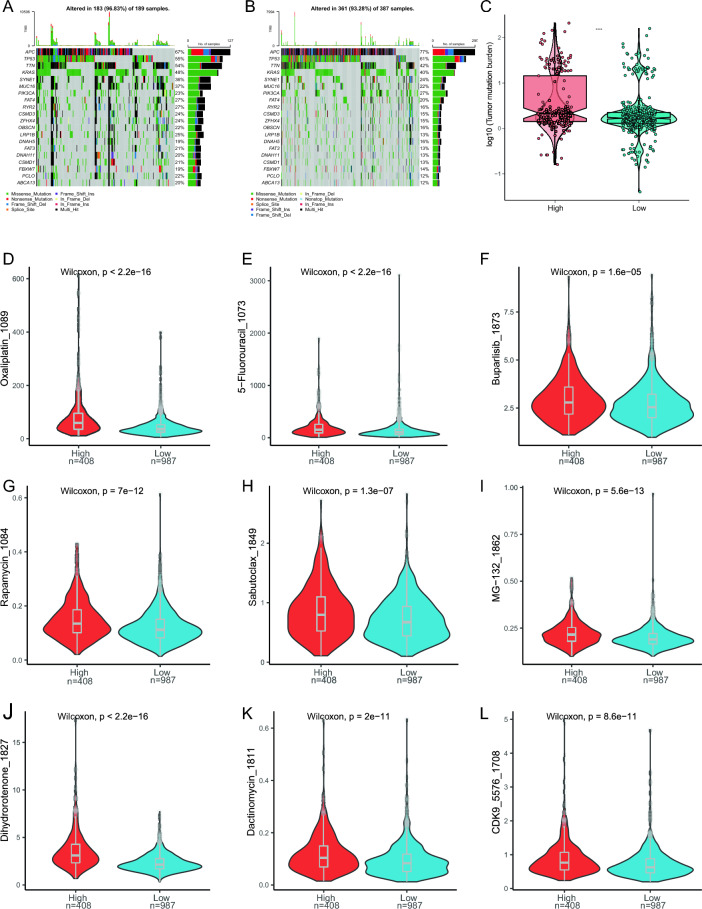
Differences in TMB and drug susceptibility between the HRG and LRG. (**A**) Top 20 genes with the highest mutation frequencies in the HRG and (**B**) LRG. (**C**) TMB difference between the two risk groups. (**D**) Difference in sensitivity to Oxaliplatin_1089 between HRG and LRG. (**E**) 5-Fluorouracil_1073. (**F**) Buparlisib_1873. (**G**) Rapamycin_1084. (**H**) Sabutoclax_1849. (**I**) MG − 132_1862. (**J**) Dihydrorotenone_1827. (**K**) Dactinomycin_1811. (**L**) CDK9_5576_1708. TMB, tumor mutation burden.

We evaluated specific gene mutations in CRC and visualized the top 20 driving genes. *AOC* has the highest mutation frequency in the two risk groups, followed by *TP53* (Fig. 10A,B). Then, we analyzed the tumor mutation burden (TMB) of somatic mutations associated with CRC, which showed that in HRG was significantly higher than LRG (*p* < 0.05) (Fig. 10C).

We analyzed whether the risk score could react to the chemosensitivity of CRC patients. The result showed patients in the LRG were sensitive to Oxaliplatin_1089 (Fig. 10D), 5-Fluorouracil_1073 (Fig. 10E), bupalisib_1873 (Fig. 10F), rapamycin_1084 (Fig. 10G), sabutoclax_1849 (Fig. 10H), Mg-132_1862 (Fig. 10I), dihydrorotenone_1827 (Fig. 10J), Dactinomycin_1811 (Fig. 10K) and CDK9_5576_ 1708 (Fig. 10L), indicating that chemotherapy was a promising option for the low-risk score group.

### Immune checkpoint analysis

**Figure 11:**
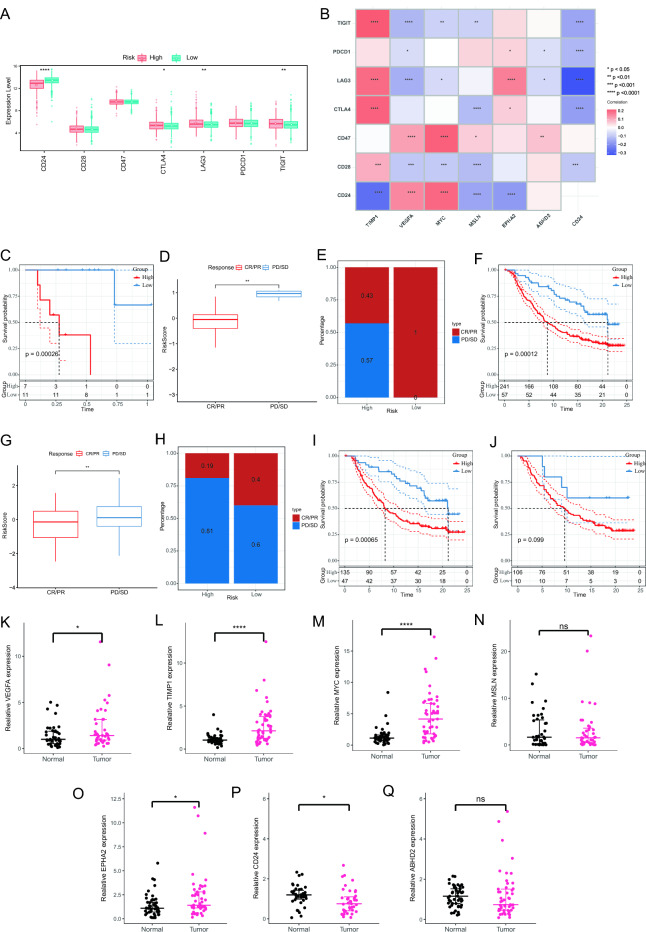
The expression pattern of immune checkpoints. (**A**) Boxplots shows estimated proportions of immune checkpoints expression between HRG and LRG, (**B**) Correlation between prognostic genes and immune checkpoints. (**C**) The survival curve of the two risk groups in the CRC immunotherapy cohort. (**D**) Differences in risk scores between patients with PR/CR and patients with PD/CD. (**E**) The High-risk group was associated with more responders (CR and PR). (**F**) The survival curve of the two risk groups in the IMvigor210 cohort. (**G**) Differences in riskscores between patients with PR/CR and patients with PD/CD. (**H**) The Low-risk group was associated with more responders (CR and PR). (**I**) The survival curve of the two risk groups of early patients in the IMvigor210 cohort. (**J**) The survival curve of the two risk groups of late patients in the IMvigor210 cohort. (**K**–**Q**) Expression of VEGFA, TIMP1, MYC, MSLN, EPHA2, CD24 and ABHD2 in CRC and normal tissues.

We investigated the ICG expression between LRG and HRG. *CD28, CD74,* and *PDCD1* show no significant differences between the two groups. CD24 is expressed at higher levels in the low-risk group, while CTLA4, LAG3, and TIGIT are expressed at higher levels in the high-risk group (Fig. 11A, Supplement Table 13). In addition, we studied the correlation between key genes and immune checkpoints, and found *TIMP1* was positively correlated with *TIGIT, LAG3, CTLA4* and *CD28*, whereas negatively correlated with *CD24*. Both *VEGFA* and *MYC* were positively correlated with *CD47* and *CD24*, with *MYC* negatively correlated with *TIGIT, LAG3* and *CD28*. *MSLN* was positively correlated with *CD47*. *EPHA2* was positively correlated with *PDCD1, LAG3* and *CTLA4*, whereas negatively with *CD24*. *ABHD2* was positively correlated with *CD47*. *CD24* was negatively correlated with *TIGIT, PDCD1, LAG3, CTLA4,* and *CD28* (Fig. 11B).

### Immunotherapy prediction

Based on our analysis of the immune microenvironment, we have discovered a strong correlation between risk models and the immune microenvironment. In order to further predict the effectiveness of immunotherapy, we acquired data from two cohorts: the colorectal cancer immunotherapy cohort and the bladder cancer immunotherapy cohort. In the colorectal cancer immunotherapy cohort, we observed a significant difference in survival time between the high-risk and low-risk groups (*p* = 0.00026) (Fig. 11C), with patients in the high-risk group experiencing notably shorter survival times. Moreover, we found that patients who exhibited a complete or partial response (CR/PR) had significantly lower risk scores compared to those who had no response or experienced disease progression (*p* < 0.01) (Fig. 11D). Interestingly, among low-risk patients, the proportion of those with a complete or partial response (CR/PR) (1.00) was much higher than among high-risk patients (0.43) after immunotherapy (Fig. 11E). To validate our findings, we conducted further analysis using the bladder cancer immunotherapy cohort. Our observations revealed that high-risk patients with bladder cancer had significantly shorter survival times compared to low-risk patients (*p* = 0.00012) (Fig. 11F). In early-stage bladder cancer patients, the high-risk group exhibited significantly lower survival times than the low-risk group (*p* = 0.00062) (Fig. 11I); however, in late-stage bladder cancer patients, there was no significant difference in survival time between the high-risk and low-risk groups (*p* = 0.099) (Fig. 11J). Additionally, we found that patients with a complete or partial response (CR/PR) had lower risk scores, while patients with no response or disease progression had higher risk scores (*p* < 0.05) (Fig. 11G). Notably, among low-risk patients, the proportion of those with a complete or partial response (CR/PR) was 0.40, whereas in the high-risk group, this proportion was 0.19 (Fig. 11H). The results from the bladder cancer cohort supported our findings, further strengthening our discoveries. These findings suggest a connection between risk models and the efficacy of immunotherapy, highlighting the potential of immunotherapy to reduce risk and improve patient survival time.

### Expression of ABHD2, TIMP1, MYC, CD24, EPHA, MSLN, and VEGFA in colorectal cancer tissues

To validate our data analysis results, we extracted total RNA from patient colon cancer tissue and corresponding normal colon epithelial tissue and measured the mRNA expression levels of *TIMP1, VEGFA, MYC, MSLN, EPHA2, ABHD2,* and *CD24* (Supplement Table 14). Through qRT-PCR testing, we observed that in colon cancer tissue, the mRNA expression levels of *VEGFA* (*p* < 0.05), *TIMP1* (*p* < 0.0001), *MYC* (*p* < 0.0001), and *EPHA2* (*p* < 0.05) were higher than in normal tissue (Fig. 11K–M,P). However, the expression of *MSLN* and *ABHD2* did not show significant differences (Fig. 11N,Q). Additionally, we also found that the expression level of *CD24* was lower in tumor tissue (*p* < 0.05) (Fig. 11O).

## Discussion

In this study, we used single-cell transcriptome data for analysis to determine the role of anoikis-related genes in regulating tumor cell development. We also identified marker genes and established prognostic prediction models to determine their value in tumor prognosis prediction. First, we analyzed the data of 5 CRC samples of single-cell sequencing dataset and identified 10 different cell types. The Anoikis gene set downloaded from the GeneCards database was used to calculate Anoikis activity using the AUCell algorithm. We identified 746 cells with Anoikis activity and found that the overall anoikis activity was higher in cells from metastatic patient samples compared to non-metastatic patients. This suggests that anoikis plays a role in regulating the occurrence and development of these tumor cells. Additionally, we established pseudo-temporal differentiation trajectories for epithelial cells, fibroblasts, and stromal cells and found that anoikis genes were mainly enriched in the late stages of cell differentiation and had different impacts on cell fate. In the pseudo-temporal analysis of mesenchymal cells, fibroblasts, and stromal cells, accompanied by an increase in anoikis activity, we observed that the gene set regulating cell fate transitioned from CXCR chemokine receptor binding and chemokine activity to structures and pathways related to the extracellular matrix (ECM). This indicates that anoikis genes play important roles in the differentiation of epithelial cells and stromal cells, as well as tumor progression. When cells escape from the ECM, these genes initiate or inhibit the anoikis program, thereby impacting the survival or death of escaping cells^38^. Therefore, these genes involved in regulating activity become key genes associated with tumor metastasis and prognosis^39^.

In order to determine the most important genes regulating anoikis activity, we compared the gene-expression difference between active and inactive cells to obtain the DEGs. We also obtained DEGs between CRC and control samples in both the single cell dataset and the bulk transcriptome dataset. After intersecting these genes, we selected ARGs from them, and obtained a total of 53 ARDEGs. These genes are not only related to anoikis, but also participate in the differentiation of CRC cells due to their differential expression, promoting the occurrence of CRC.

We tried to annotate the functions and pathways of these genes and found that they are enriched in cell substrate adhesion, cell matrix adhesion, protein phosphatase binding, collagen-containing extracellular matrix, PI3K-Akt signaling pathway, cancer proteoglycan and other functions and pathways. These pathways are all related to the adhesion of cells to the ECM, which ensures the normal activity of cells. However, once these adhesion related genes are suppressed in CRC tissues and anoikis activated cells, tumor cells may escape the ECM and become anoikis resistant, leading to tumor invasion and metastasis.

We attempted to identify prognostic related genes from the 53 ARDEGs. Through UCRA and LASSO, we identified 7 characteristic genes *TIMP1, VEGFA, MYC, MSLN, EPHA2, ABHD2,* and *CD24*. In addition, we further verified that *VEGFA, TIMP1, MYC,* and *EPHA2* were significantly highly expressed in tumor tissues. *TIMP1* encodes an inhibitor protein related to Matrix metalloproteinase (MMPs), participating in the degradation of extracellular matrix, promoting the proliferation of tumor cells, and has the function of anti-anoikis^40^. In the study by Yang et al.^41^, *TIMP1*, as a characteristic gene of CRC, could predicts prognosis of the cancer, which is consistent with the result of this study. VEGFA encodes a growth factor within the VEGF family, which is a glycosylated protein specifically present in endothelial cells. It promotes cell growth and differentiation and inhibits apoptosis^42^. *MYC* encodes a nuclear phosphoprotein with the function of transcription factor and participates in cell cycle regulation. Its abnormal expression and gene mutation have been confirmed to be related to lymphoma, leukemia and other diseases. Wu QN et al. study^43^ showed that *MYC* participates in the MYC-MNX1-AS1-YB1 axis, which promotes the proliferation of colorectal cancer cells. MSLN overexpression promotes cell migration and invasion through the activation and expression of matrix metalloproteinases, specifically MMP-7 and MMP-9^44,45^. Furthermore, the high-affinity interaction between MSLN and CA125 leads to heterotypic cell adhesion, thereby promoting metastasis of ovarian cancer cell lines^46^. EPHA2 has been demonstrated to regulate multiple cellular processes in embryonic development, angiogenesis, and tumor occurrence through EphA2-ephrin A1 signaling transduction, including proliferation, survival, migration, morphology, cell repulsion, and adhesion^47^. ABHD2 is a novel androgen-regulated gene that can enhance prostate cancer growth and chemotherapy resistance^48^. CD24, a cell surface molecule linked to glycosylphosphatidylinositol, is considered an adhesion molecule that facilitates binding with P-selectin and is a characteristic of cancer metastasis.

We constructed a prognosis prediction model based on these 7 characteristic genes, which has good predictive ability. We used this model to perform prognostic risk scoring on all CRC samples and divided them into LRG and HRGs. In the analysis of GSEA and GSVA of the groups, enrichment pathways such as ECM receptor ligand, focal adhesion, pyruvic acid metabolism, and peroxisome pathway were found. The interaction and adhesion pathway of ECM receptor ligands are key pathways for tumor cells to escape their original survival environment and initiate re colonization. Pyruvic acid and peroxisome also participate in the anti-anoikis of tumor cells. Giannoni et al.^49^ found a strong correlation between the oxidative environment inside the envelope of cancer cells and the induction of anti-anoikis. Compared with normal cells, the glucose metabolism of tumor cells has undergone fundamental changes. The pyruvic acid salt produced by glycolysis is converted into lactic acid instead of entering the mitochondrial oxidation pathway, thus avoiding the excess reactive oxygen species (ROS) produced by cells and changing the oxidative environment in cells. After detachment from the matrix, these tumor cells could reduce glucose oxidation and utilize their survival advantages to resist anoikis.

To further analyze the prognostic predictive value of this model, we included the clinical characteristics of patients, and together with the prognostic risk score, we constructed a nomogram for predicting patient survival. ROC analysis showed that the nomogram has high predictive value for patients, with AUC exceeding 0.8 for 1 year, 3 years, and 5 years.

In the analysis of immune infiltration between the risk groups, we found that activated B cells, CD4 T cells, and CD8 T cells were more expressed in the HRG, while dendritic cells and NK cells was less. B cells could promote the formation of immunosuppressive cells by releasing immunosuppressive cytokines, and also participate in the process of presenting tumor antigens to CD4 T and CD8 T cells, directly attacking tumor cells through Granzyme B, and are important anti-tumor cells^50^. T cells are specific tumor killer cells that can secrete TNF- α Suppress the growth of tumor cells^51^. The immune cell infiltration of these cells might lead to poor prognosis in high-risk patients. We also found the 7 characteristic genes were corelated with the immune infiltration, such as *MYC* is associated with the expression of NK cells and B cells, while *TIMP1* is significantly associated with the expression of *CD* 8 T cells, macrophages, Mast cell, Monocyte, Natural killer cell and regulatory T cells. We speculated that *MYC* and TIMP1 are involved in the occurrence and development of CRC by regulating the infiltration of these immune cells, and these two genes might also become targets for immunotherapy.

Based on the correlation between characteristic genes and immune infiltration, we attempt to infer the sensitivity of immunotherapy in patients at different risk groups. We investigated the expression of ICGs in the two groups and found that most ICGs highly expressed in the HRG, Furthermore, we further utilized immune therapy cohorts for colorectal cancer and bladder cancer to predict treatment outcomes. We found that high-risk patients had shorter survival times in both cohorts. Additionally, we observed that patients who achieved complete response (CR) and partial response (PR) through immune therapy had lower risk scores. Moreover, the proportion of patients who achieved complete response (CR) and partial response (PR) among low-risk patients receiving immune therapy was much higher much higher than among high-risk patients. Colorectal cancer (CRC) has an extremely complex tumor microenvironment (TME). The effectiveness of immunotherapy is influenced by various factors. Immune suppressive cells in the TME, such as regulatory T cells (Tregs), myeloid-derived suppressor cells (MDSCs), and tumor-associated macrophages (TAMs), can inhibit the activity of T cells by secreting immune-suppressive factors or directly interacting with effector T cells, thereby reducing the effectiveness of immunotherapy^52^. Additionally, the extracellular matrix (ECM) and vascular structure in the TME also impact the infiltration and function of immune cells. Tumor cells remodel the ECM and vascular system to create an environment that supports tumor growth and metastasis, while potentially limiting effective infiltration and anti-tumor activity of immune cells^52^. In our study, we observed a higher infiltration of activated CD8 + T cells and NK cells in the low-risk group. Therefore, the effectiveness of immunotherapy in colorectal cancer patients may be more influenced by the presence of tumor-infiltrating immune cells with cytotoxic activity against the tumor. These results suggest that patients with a low-risk score may benefit more from treatment with ICG inhibitors. The results of drug sensitivity prediction analysis showed that patients in the LRG might be more sensitive to the treatment of oxaliplatin, 5-fluorouracil, Bupaleb, Rapamycin, Sabutoclax, MG-132 and other chemotherapy drugs, suggesting that chemotherapy is a better choice for patients with low-risk scores.

In the study by Xiao et al.^53^, the LASSO regression method was also used to identify 25 ARGs related to CRC prognosis, which is completely different from the 7 genes obtained in this study. In their study, 540 CRC sample from TCGA were selected, whereas in this study, we selected dataset from both TCGA and GEO. We also included the single cell sequencing data and bulk size of RNA seq data, thus the conclusions of this study are more reliable.

But there are also some limitations in this study. First, we identified specific gene signature from the gene database wihout confirming their predictive significance for prognosis in large-scale prospective clinical studies; Second, some of the conclusions in our study were still based on inference and there was no clear interaction pathway to support them, such as the regulatory relationship between *MYC* and immune cell expression; Third, we have developed a highly accurate prognostic prediction model, but the application value of this model still needs to be proven in practical use.

## Conclusions

In this study, based on single cell sequencing data and bulk transcriptome dataset of public databases we found that the anoikis-related genes play an important role in the development and metastasis of CRC tumor cells. We obtained 7 marker genes related to the prognosis of CRC and constructed a prognosis prediction model, which has high accuracy in predicting prognosis. Based on this model, the tumor samples could be divided into different risk groups, with different sensitivities to chemotherapy drugs and immune checkpoint inhibitors. This study is expected to provide new insights into the precision diagnosis and treatment of CRC.

### Supplementary Information

Supplementary Figures.

Supplementary Table 1.

Supplementary Table 2.

Supplementary Table 3.

Supplementary Table 4.

Supplementary Table 5.

Supplementary Table 6.

Supplementary Table 7.

Supplementary Table 8.

Supplementary Table 9.

Supplementary Table 10.

Supplementary Table 11.

Supplementary Table 12.

Supplementary Table 13.

Supplementary Table 14.

## Data Availability

The datasets used in this study are available in TCGA (https://portal.gdc.cancer.gov/), GEO (https://www.ncbi.nlm.nih.gov/geo/), and TIDE databases (http://tide.dfci.harvard.edu/). IMvigor210 database was downloaded by the IMvigor210CoreBiologies R package (http://research-pub.gene.com/IMvigor210CoreBiologies/packageVersions/). The datas generated and analysed during the current study are available from the the article and supplementary information.

## Abbreviations

CRC: Colorectal cancer
TCGA: The cancer genome atlas
GEO: Gene expression omnibus
DEGs: Differentially expressed genes
ARDEGs: Anoikis-related differentially expressed genes
UCRA: Univariate cox regression analysis
MCRA: Multivariate cox regression analysis
HRG: High risk group
LRG: Low risk group
TMB: Tumor mutational burden
ICG: Immune checkpoint genes
PR: Partial response
CR: Complete response
PD: Progressive disease
SD: Stable disease
