# Supplementary material for: Anoikis-related gene signatures in colorectal cancer: implications for cell differentiation, immune infiltration, and prognostic prediction
Source: Sci Rep. 2024 May 21;14:11525. doi: 10.1038/s41598-024-62370-y (PMC11109202; doi:10.1038/s41598-024-62370-y)
Supplement: Supplementary file 1 — Supplementary Figures. [file 41598_2024_62370_MOESM1_ESM.docx]

**
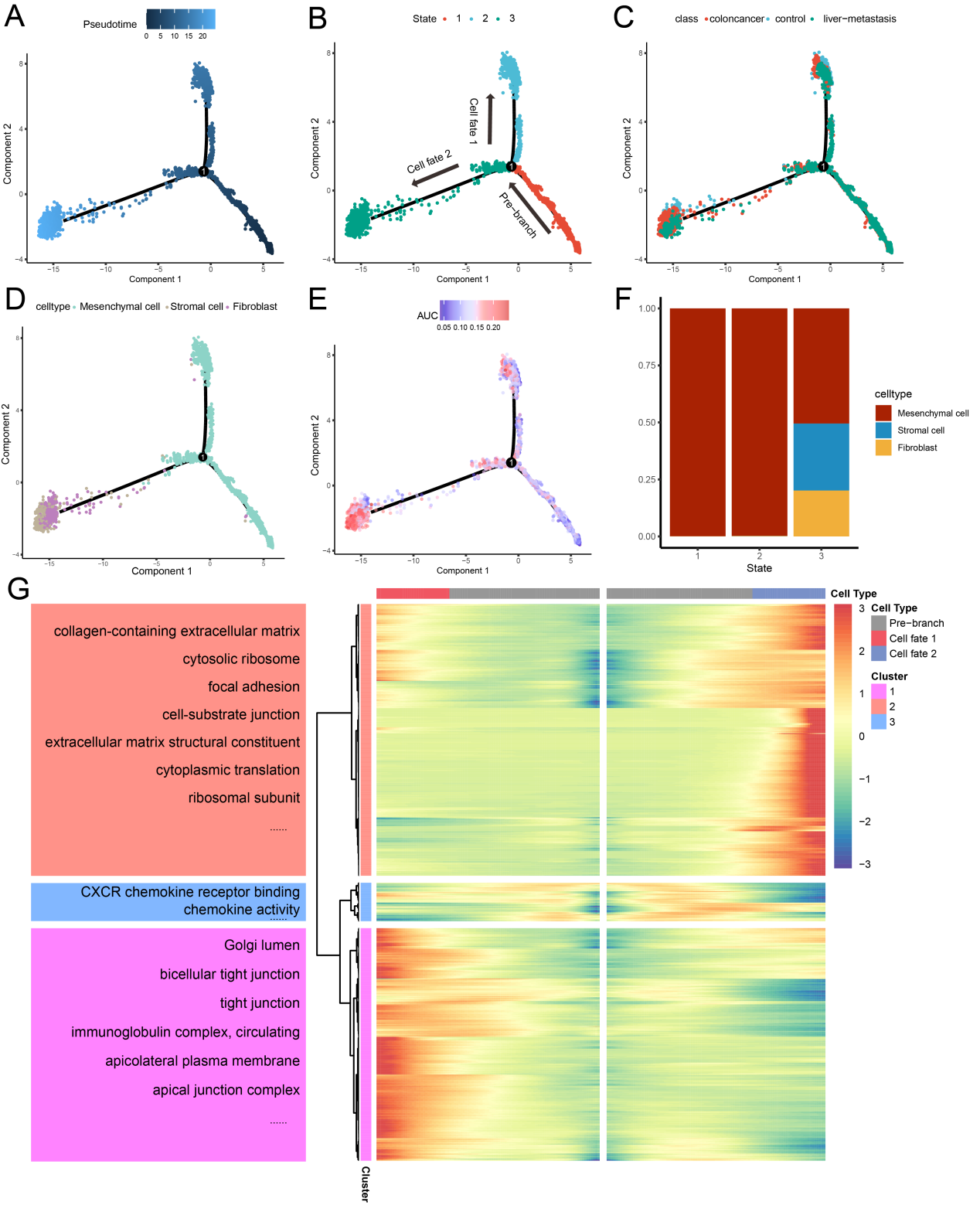
 Figure. S1 Transcriptional trajectory analysis revealed an association between anoikis and differentiation of Mesenchymal cells into fibroblasts and stromal cells. (A) The pseudo-time gradient transitions. (B) Three different states of the trajectory. (C) Pseudotime plots of different class. (D) Pseudotime plots of different celltype. (E) Stacked bar graphs of cell types in different states. (F) The DEGs of branches (fates) show in heatmap. DEGs, differentially expression genes.**

**
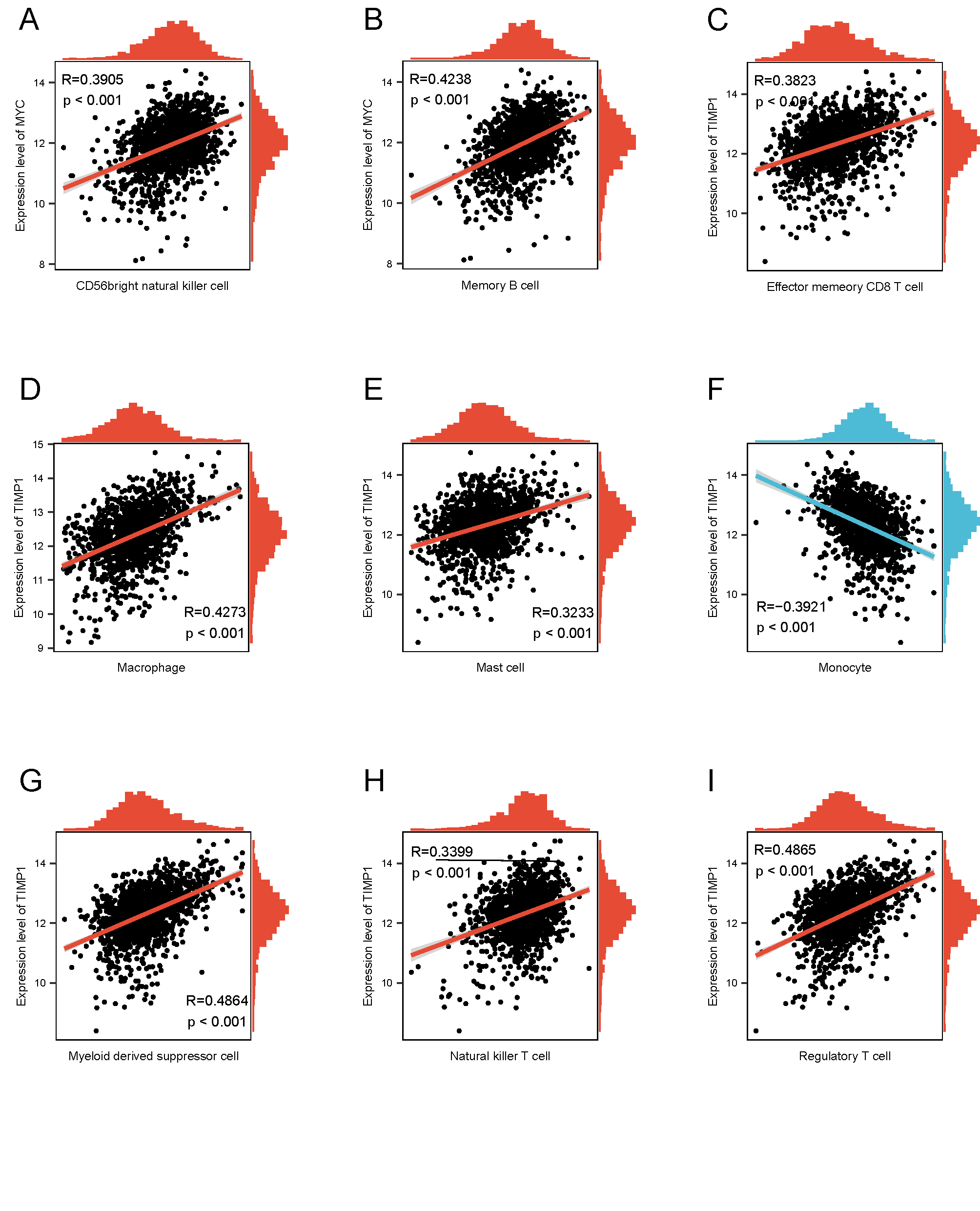
**

**Figure. S2 Correlation between immune cells and genes. Correlation of gene MYC with CD56bright natural killer cell (A) and Memory B cell (B); Correlation of TMP1 and Effector memeory CD8 T cell (C), Macrophage (D), Mast cell (E), Monocyte (F), Myeloid derived suppressor cell Correlation of (G), Natural killer cell (H), and Regulatory T cell (I).**
